# Preoperative predictors of poor outcomes in Thai patients with aneurysmal subarachnoid hemorrhage

**DOI:** 10.1371/journal.pone.0264844

**Published:** 2022-03-15

**Authors:** Punnarat Sirataranon, Pichayen Duangthongphon, Phumtham Limwattananon

**Affiliations:** Neurosurgery Unit, Department of Surgery, Faculty of Medicine, Khon Kaen University, Khon Kaen, Thailand; University of Florida, UNITED STATES

## Abstract

**Objective:**

A scoring system for aneurysmal subarachnoid hemorrhage (aSAH) is useful for guiding treatment decisions, especially in urgent-care limited settings. This study developed a simple algorithm of clinical conditions and grading to predict outcomes in patients treated by clipping or coiling.

**Methods:**

Data on patients with aSAH hospitalized in a university’s neurovascular center in Thailand from 2013 to 2018 were obtained for chart review. Factors associated with poor outcomes evaluated at one year were identified using a stepwise logistic regression model. For each patient, the rounded regression coefficients of independent risk factors were linearly combined into a total score, which was assessed for its performance in predicting outcomes using receiver operating characteristic analysis. An appropriate cutoff point of the scores for poor outcomes was based on Youden’s criteria, which maximized the summation between sensitivity or true positive rate and the specificity or true negative rate.

**Results:**

Patients (n, 121) with poor outcomes (modified Rankin Scale, mRS score, 4–6) had a significantly higher proportion of old age, underlying hypertension, diabetes and chronic kidney disease, high clinical severity grading, preoperative rebleeding, and hydrocephalus than those (n, 336) with good outcomes (mRS score, 0–3). Six variables, including age >70 years, diabetes mellitus, World Federation of Neurosurgical Societies (WFNS) scaling of IV-V, modified Fisher grading of 3–4, rebleeding, and hydrocephalus, were identified as independent risk factors and were assigned a score weight of 2, 1, 2, 1, 3 and 1, respectively. Among the total possible scores ranging from 0–10, the cut point at score 3 yielded the maximum Youden’s index (0.527), which resulted in a sensitivity of 77.7% and specificity of 75.0%.

**Conclusion:**

A simple 0–10 scoring system on six risk factors for poor outcomes was validated for aSAH and should be advocated for use in limited resource settings.

## Introduction

Aneurysmal subarachnoid hemorrhage (aSAH) accounts for 5–10% of hemorrhagic strokes and is associated with a high mortality rate [1–3]. Ten percent of these cases may die before reaching a hospital [4], and a third will remain disabled despite treatment [5]. The mortality rates are the highest within the first year after treatment [6, 7]. In the long term, patients with aSAH have excess mortality compared with the general population [8].

Several studies have identified factors affecting functional dependency or mortality in patients with aSAH [9–12]. Predicting the outcomes of the disease contributes insightful guidance for clinicians during treatment decision-making. It can provide invaluable information for patients and relatives in navigating the treatment or no-treatment dilemma. Regarding measures of clinical severity, the most commonly used measurement is the World Federation of Neurosurgical Societies (WFNS) scaling system [13]. The WFNS scale, which is measured after adequate neurological resuscitation, is a good predictor of treatment outcomes [14, 15]. Preoperative rebleeding was found to be a risk factor for short-term mortality [16, 17]. Other factors associated with poor outcomes include old age, hyperglycemia, hydrocephalus, and high-grade Fisher scores [1, 2, 4, 9, 18].

Model-based scoring systems using the aforementioned risk factors have been used to predict outcomes in patients with aSAH. Three scoring systems were recently developed for patients who were mostly treated with microscopic clipping or endovascular coiling [9–11]. Despite differences in patient characteristics and clinical conditions, the outcome predictors and their scoring weights reported across studies were quite similar. The first study published in 2006 was conducted in a small number of patients with poor grades (Hunt-and-Hess grade of 4–5) [9]. The second study in 2019 was conducted in patients mostly with good grades (WFNS scale of I-II), even though 60% of the patients had high Fisher scores [10]. The most recent study in 2021 was conducted in patients with a Fisher score of 3 [11]. Access to aneurysmal treatment in Thailand is limited to certain health care facilities, and only a few university-level hospitals have the resources to perform both microscopic clipping and endovascular coiling [6, 19–21]. Due to the differences in patient and treatment profiles, it is worthwhile for Thailand to advocate a scoring system that could be applied to a wide range of patients with aSAH at various levels of health care.

The aim of this study was to identify factors independently affecting poor outcomes at one year after onset in patients with aSAH who received treatment. A scoring system was developed from a predictive model using patient characteristics at the time of presentation to the hospital prior to definite treatment.

## Materials and methods

### Ethics approval

The study was approved by the Center for Ethics in Human Research, Khon Kaen University (HE621167). The institutional ethics committee waived the requirement for informed consent due to a retrospective nature to obtain data from medical records. All data were fully anonymized throughout the study process.

### Study settings and patients

This retrospective study was conducted in a university’s neurovascular center that admitted patients typically referred from lower-level facilities located in the northeastern region of Thailand. The study site has a high volume of microscopic clipping and endovascular coiling. All patients presenting with aSAH were treated according to the center’s standard protocol [6] and neurovascular care was provided by trained nurses. aSAH was confirmed by either computerized tomographic angiogram (CTA) or magnetic resonance angiography (MRA) or by cerebral digital subtraction angiography (DSA) if necessary. All patients with a Glasgow Coma Score (GCS) [22] of less than 8 were intubated. Patients’ systolic blood pressures (SBPs) were controlled within the range of 120–160 millimeters of mercury (mmHg). Patients with symptomatic hydrocephalus had extraventricular drains inserted to control their intracranial pressure within 20–25 centimeters of water column at 4 degC (cmH_2_O). All patients received oral nimodipine and intravenous antiepileptic drugs as clinically indicated. The choice of aneurysmal obliteration was discussed with an interdisciplinary team and the patient or relatives. Aneurysmal treatment was implemented as soon as possible, usually within 48 hours after hospital admission or 72 hours after onset. Outpatient follow-up was pursued at 1, 3, 6 and 12 months plus yearly afterward. At each follow-up visit, the modified Rankin Score (mRS) [23] of each patient was assessed by the neurosurgeons. Patients who did not attend their visits as planned were reached for telephone interviews by trained nurses.

Patients diagnosed with CTA-, MRA- or DSA-confirmed aSAH from 2013 to 2018 who received definite treatment by either clipping or coiling were included in this study. Patients with aSAH due to traumatic aneurysms, mycotic aneurysms or arteriovenous malformations were excluded.

### Study variables

The primary endpoint was poor outcomes after definite treatment, which was indicated by the combined dependency or death at one year after treatment. A dependent state was defined as an mRS score of 4–5 (in contrast with an independent state with an mRS score of 0–3), whereas an mRS score of 6 meant death.

Potential risk factors were obtained from a comprehensive review of inpatient and outpatient medical records by a well-trained nurse at the study center. The retrieved data covered patient demographics, including age and sex, and health risks, including overweight defined internationally by the World Health Organization as a body mass index of 25 kilograms per square meter (kg/m^2^) or over, and the history of smoking and alcohol consumption. Clinical risk factors were the presence of underlying diseases, including hypertension, diabetes mellitus (DM), and chronic kidney disease. High SBP was indicated by a blood pressure measurement at initial admission of over 160 mmHg. Preoperative neurological values after neurological resuscitation were recorded using WFNS scaling and GCS. The scaling of WFNS I-III was defined as good grading and that of WFNS IV-V was defined as poor grading. Radiologic variables included hydrocephalus, modified Fisher (mFisher) grading [24], aneurysmal location and size, and the number of aneurysms. Hydrocephalus was defined according to Evan’s ratio with 0.3 as the cut point [25]. The aneurysmal location was categorized as anterior cerebral artery, internal carotid artery, middle cerebral artery, and vertebrobasilar artery. The anterior cerebral artery is comprised of the anterior communicating artery and proximal and distal anterior cerebral arteries. The internal cerebral artery consists of the posterior communicating artery, anterior choroidal artery, and ophthalmic artery. Aneurysms of the posterior cerebral artery, superior cerebellar artery, anterior inferior cerebellar artery, posterior inferior cerebellar artery, vertebral artery and basilar artery were considered vertebrobasilar arteries. All radiologic variables were based on the initial neuroimaging obtained. Rebleeding was defined as a new episode of CT-confirmed, aSAH-related hemorrhage prior to treatment. The time to definite treatment was calculated from the aSAH onset to the intervention given, either surgical or endovascular.

### Statistical analysis

To summarize patient characteristics and clinical conditions, continuous variables are presented as the mean and standard deviation (SD), and categorical variables are presented as the frequency and percentage. Each risk factor was compared between patient subgroups, either poor or good outcomes for statistical significance at p <0.05, using an independent t-test for the mean difference or Pearson’s chi-square test for the proportion difference. Comparing all-cause mortality of patients across age groups and the WFNS scale and mFisher scaling was performed using Kaplan–Meier estimates.

Factors associated with a poor outcome were determined using logistic regression, and the magnitude of the associations was measured in terms of an odds ratio (OR) with a 95% confidence interval (CI). A univariate analysis of each risk factor was performed, yielding an unadjusted OR to guide predictors of the poor outcome. Multivariate analysis was initiated with the full model containing all potential risk factors. A stepwise approach was undertaken to achieve a final model using backward elimination with a statistical criterion at p ≥0.05 for removal of variables. The performance of the model containing retained risk factors was determined by the area under the receiver operating characteristic (ROC) curve, in which values of 0.50–0.59, 0.60–0.69, 0.70–0.79, 0.80–0.89 and 0.90–1.00 were interpreted as no, poor, acceptable, excellent and outstanding, respectively, in discriminating between binary outcomes [26, 27].

Predicted scores of the poor outcome were calculated as a linear combination of a regression coefficient (or log OR) of each predictor retained in the final model, excluding the constant term. A predictor with a positive signed coefficient (or OR >1.0) was a risk factor for a poor outcome, whereas a negative coefficient (or OR <1.0) represented a preventive factor. For practical and clinical applicability reasons, coefficients of 0.51–1.50, 1.51–2.50, 2.51–3.50, … were assigned scores of 1, 2, 3, … to an individual patient presenting with the risk factors.

The ability of a given score cutoff to correctly detect patients who had a poor outcome in contrast to those with a good outcome was a tradeoff. In our case, using the cutoff at a score of 0 would effectively cover all patients with poor outcomes (sensitivity, 100%), but it would miss all patients with good outcomes (specificity, 0% or false-positive rate, 100%). An increase in the cutoff to scores of 2, 3, 4, … would lower the sensitivity while raising the specificity. Youden’s index that maximized the true positive rate (or sensitivity) and minimized the false-positive rate (or 1 –specificity) and was equal to “sensitivity + specificity– 1” was used for selecting the appropriate cutoff over a possible score range of the study patients [28].

## Results

### General characteristics

A total of 519 patients with aSAH were admitted during the study period. Sixty-two patients were excluded as they received neither microvascular clipping nor endovascular coiling. Among 457 patients included and analyzed in the present study, 14 (3.1%) had an in-hospital death and 65 (14.2%) died within one year after hospitalization. There were no losses to follow up among 378 patients who were the survivors at one year. Seventeen (4.5%) and 25 (6.6%) patients had a poor mRS score of 4 and 5, respectively; whereas 134 (35.4%), 121 (32.0%), 46 (12.2%) and 35 (9.3%) patients had a good mRS score of 0, 1, 2 and 3, respectively.

The mean age of the study patients was 54.5 years, 39.8% were male, 14.0 and 10.3% were smokers and alcohol drinkers, respectively, and 17.5% were obese (Table 1). More than half (52.7%) of the study patients had underlying hypertension, 10.3% had DM and 2.6% had chronic kidney disease. Aneurysmal rupture was mostly located in the anterior cerebral artery (45.2%), followed by the internal carotid artery (28.7%), middle cerebral artery (15.1%), and vertebrobasilar artery (11%). Almost half (46.1%), 37.3%, 13.6%, 2.9% and 0.2% of patients had aneurysm sizes of 0–4.0, 4.1–7.0, 7.1–13.0, 13.1–25.0 or larger than 25.0 millimeters (mm), respectively. Approximately 7% had nonsaccular aneurysms.

**Table 1:** Demographic and medical characteristics.

	Overall, n (%)	Good outcome, n (%)	Poor outcome, n (%)	p value^a^
Total patients	457 (100.0)	336 (73.5)	121 (26.5)	
Age in years, mean *±* SD	54.5±12.4	52.5±11.3	60.2±13.6	<0.001
Age >70 years	43 (9.4)	15 (4.5)	28 (23.1)	<0.001
Male	182 (39.8)	139 (41.4)	43 (35.5)	0.261
Smoking	64 (14.0)	47 (14.0)	17 (14.1)	0.987
Alcohol drinking	47 (10.3)	34 (10.1)	13 (10.7)	0.846
Overweight	79 (17.5)	58 (17.4)	21 (17.7)	0.955
Hypertension	241 (52.7)	166 (49.4)	75 (62.0)	0.017
Diabetes mellitus	47 (10.3)	22 (6.5)	25 (20.7)	<0.001
Chronic kidney disease	12 (2.6)	5 (1.5)	7 (5.8)	0.011
Initial SBP >160 mmHg	114 (25.0)	77 (23.0)	37 (30.6)	0.098
Initial GCS, mean ± SD	12.7±3.2	13.5±2.7	10.6±3.4	<0.001
WFNS grading				<0.001
I	231 (50.5)	207 (61.6)	24 (19.8)	
II	59 (12.9)	44 (13.1)	15 (12.4)	
III	17 (3.7)	15 (4.5)	2 (1.7)	
IV	115 (25.2)	53 (15.7)	62 (51.2)	
V	35 (7.7)	17 (5.1)	18 (14.9)	
mFisher grading				<0.001
1	45 (9.8)	43 (12.8)	2 (1.7)	
2	45 (9.8)	41 (12.2)	5 (4.1)	
3	215 (47.1)	159 (47.3)	56 (46.3)	
4	151 (33.0)	93 (27.7)	58 (47.9)	
Time to intervention, hours				0.214
<24	31 (6.8)	19 (5.7)	12 (9.9)	
24–72	161 (35.2)	123 (36.6)	38 (31.4)	
>72	265 (58.0)	194 (57.7)	71 (58.7)	
Location of the ruptured aneurysm				0.506
Anterior cerebral artery	206 (45.2)	148 (44.1)	58 (48.3)	
Internal carotid artery	131(28.7)	95 (28.3)	36 (30.0)	
Middle cerebral artery	69 (15.1)	52 (15.5)	17 (14.2)	
Vertebrobasilar artery	50 (11.0)	41 (12.2)	9 (7.5)	
Aneurysm size, millimeters				0.934
0–4.0	210 (46.1)	158 (47.0)	53 (43.8)	
4.1–7.0	170 (37.3)	123 (36.6)	47 (38.9)	
7.1–13.0	62 (13.6)	45 (13.4)	17 (14.0)	
3.1–25.0	13 (2.9)	9 (2.7)	4 (3.3)	
>25.0	1 (0.2)	1 (0.3)	0 (0)	
Multiple aneurysms	68 (14.9)	45 (13.4)	23 (19.0)	0.137
Nonsaccular aneurysm	33 (7.2)	25 (7.4)	8 (6.6)	0.763
Rebleeding from aneurysm	11 (2.4)	2 (0.6)	9 (7.4)	<0.001
Hydrocephalus	188 (41.1)	117 (34.8)	71 (58.7)	<0.001
Surgical clipping	421 (92.1)	307 (91.4)	114 (94.2)	0.319
Endovascular coiling	36 (7.9)	29 (8.6)	7 (5.8)	

^a^ Pearson’s chi-square test, except for the independent t-test for age and initial GCS.

GCS, Glasgow coma score; mFisher, modified Fisher; mmHg, millimeters of mercury; n, number; SAH, subarachnoid hemorrhage; SBP, systolic blood pressure; SD, standard deviation; WFNS, World Federation of Neurosurgical Societies.

Patients with poor outcomes (mRS score, 4–6; n, 121) were significantly older and had significantly more underlying diseases than those with good outcomes (mRS score, 0–3; n, 336) (Table 1). Furthermore, the poor outcome group had a significantly higher proportion of high-grade WFNS, high-grade mFisher, preoperative rebleeding, and hydrocephalus than the good outcome group. A higher proportion among those with poor outcomes had a higher initial SBP, larger (>4 mm) size of the aneurysm, multiple aneurysms, and time to interventions <24 hours than those with good outcomes but they did not reach statistical significance (p>0.05). An initial GCS in the poor outcome group on average seemed to be lower than that in the good outcome group.

Most (92.1%) patients received microsurgical clipping, and 7.9% received endovascular coiling. Among 121 patients with poor outcomes, 69 died from aSAH within 1 year after the treatments. None of the patients in the good outcome group died within 1 year.

Over the median follow-up of 726 days, 94 patients died from any cause (88 and 6 in the poor and good outcome groups, respectively), and 82 died from aSAH (81 and 1 in the poor and good outcome groups, respectively). Comparing across age groups, WFNS scaling and mFisher scores, patient survival in those >70 years, WFNS IV-V, and mFisher 3–4 were lower than their counterparts (S1–S3 Figs).

### Factors associated with treatment outcomes at one year

When the patient’s demographic and clinical characteristics were analyzed one at a time for an association with treatment outcomes, age >70 years, presence of underlying hypertension, DM and chronic kidney disease, having a high grade of WFNS (IV-V) and mFisher (3–4), an experience of preoperative rebleeding and having hydrocephalus increased the likelihood of poor outcomes (OR, 6.44, 1.67, 3.72, 4.06, 7.15, 5.43, 13.42 and 2.66, respectively; p <0.05; Table 2, Columns 2 and 3). When all characteristics were taken simultaneously for a multivariable analysis, the factors remaining statistically associated with the treatment outcomes in the same model included age >70 years, DM, WFNS (IV-V), mFisher (3–4), rebleeding and hydrocephalus (OR, 5.16, 2.48, 5.30, 3.68, 18.00 and 2.22, respectively; p <0.05; Table 2, Columns 4 and 5).

**Table 2:** Factors associated with a poor outcome at one year.

Variable	Univariate analysis		Multivariate analysis			
			Full model		Final model	
	Odds ratio	95% CI	Odds ratio	95% CI	Odds ratio	95% CI
Age >70 years	6.44**	3.30–12.57	5.16**	2.30–11.59	5.28**	2.41–11.57
Male	0.78	0.51–1.20	0.84	0.45–1.59		
Smoking	1.01	0.55–1.83	1.24	0.49–3.16		
Alcohol drinking	1.07	0.54–2.10	1.72	0.66–4.45		
Overweight	1.02	0.59–1.76	1.16	0.59–2.28		
Hypertension	1.67*	1.09–2.55	1.30	0.75–2.27		
Diabetes mellitus	3.72**	2.01–6.89	2.48*	1.11–5.56	2.92**	1.40–6.11
Chronic kidney disease	4.06*	1.27–13.06	2.79	0.59–13.15		
Initial SBP >160 mmHg	1.48	0.93–2.34	1.18	0.64–2.16		
WFNS grading (IV–V vs. I-III)	7.15**	4.53–11.31	5.30**	3.09–9.09	5.40**	3.23–9.05
mFisher grading (3–4 vs. 1–2)	5.43**	2.43–12.11	3.68**	1.47–9.23	3.30**	1.36–7.96
Time to intervention, hours						
<24	1		1			
24–72	0.49	0.22–1.10	0.71	0.27–1.89		
>72	0.58	0.27–1.25	1.02	0.39–2.64		
Posterior circulation location	0.77	0.42–1.43	0.48	0.19–1.18		
Aneurysm size >4 mm	1.13	0.74–1.72	1.05	0.62–1.77		
Multiple aneurysms	1.52	0.87–2.64	0.96	0.46–1.99		
Nonsaccular aneurysm	0.88	0.39–2.01	1.44	0.44–4.76		
Rebleeding from aneurysm	13.42**	2.86–63.04	18.00**	3.16–102.47	17.70**	3.25–96.43
Hydrocephalus	2.66**	1.74–4.07	2.22**	1.29–3.81	1.95*	1.17–3.23

* p <0.05

** p <0.01.

CI, confidence interval; mFisher, modified Fisher; mm, millimeters; mmHg, millimeters of mercury; SBP, systolic blood pressure; WFNS, World Federation of Neurosurgical Societies.

The final model of factors retained as the predictors of poor outcomes covered the following: age >70 years, presence of DM, WFNS IV-V, mFisher 3–4, rebleeding and presence of hydrocephalus (OR, 5.28, 2.92, 5.40, 3.30, 17.70 and 1.95, respectively; p <0.05; Table 2, Columns 6 and 7).

### Model-based scores predicting poor outcomes

Each predictor was assigned an appropriate scoring weight based on regression coefficients of the final model. The score of each factor was 2, 1, 2, 1, 3 and 1 for age >70 years, presence of DM, WFNS IV-V, mFisher 3–4, rebleeding and presence of hydrocephalus, respectively (Table 3), and resulted in a total possible score between 0 and 10.

**Table 3:** Scoring system for the prediction of poor outcome at one year.

Variable	Coefficient^a^	Weight
Age > 70 years	1.66**	2
Diabetes mellitus	1.07**	1
WFNS grading (IV-V vs. I-III)	1.69**	2
mFisher grading (3–4 vs. 1–2)	1.19**	1
Rebleeding aneurysm, preoperative	2.87**	3
Hydrocephalus	0.67*	1

^a^ based on a logistic regression.

* p <0.05

** p <0.01.

mFisher, modified Fisher; WFNS, World Federation of Neurosurgical Societies.

**Fig 1:**
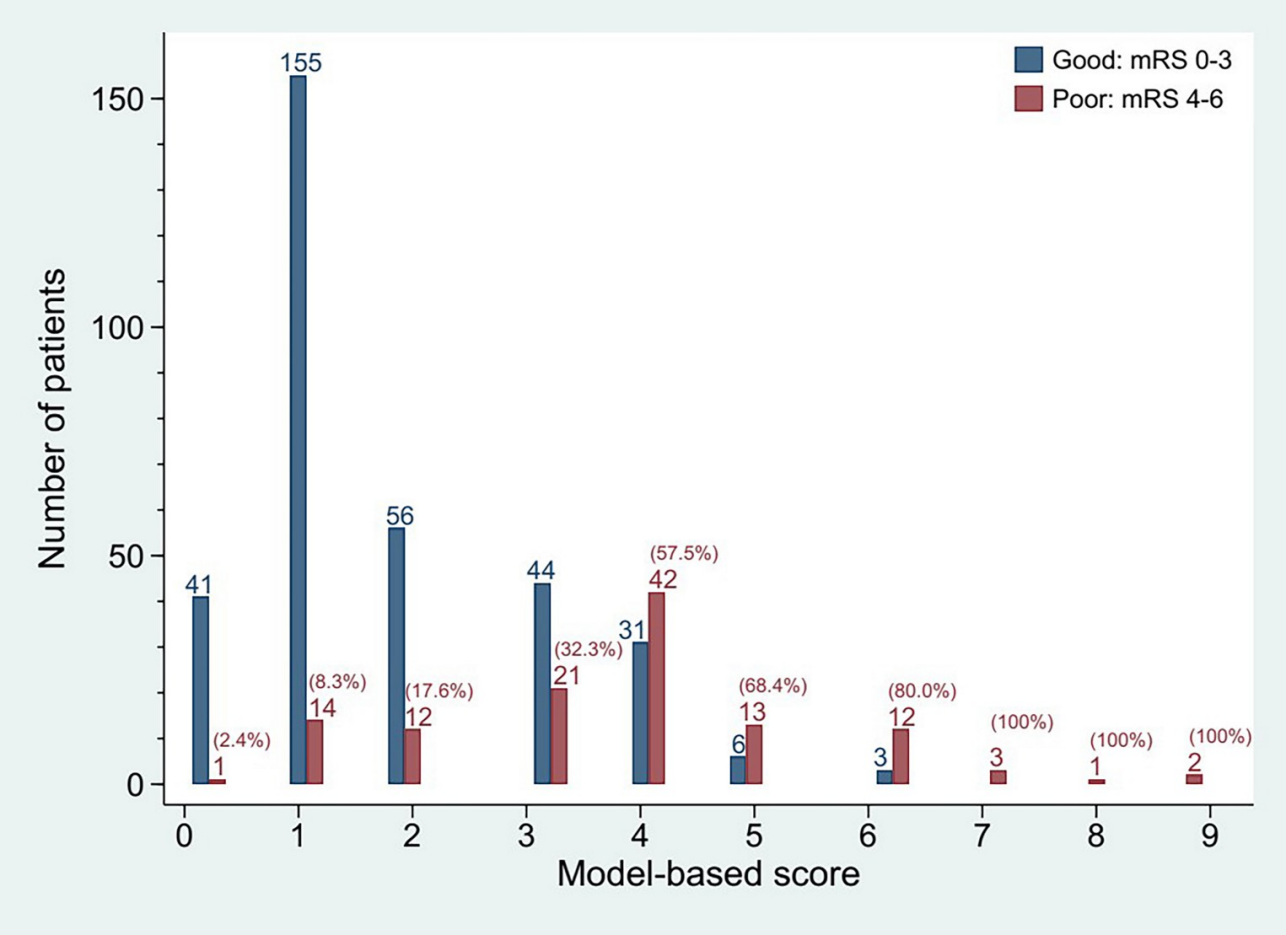
Distribution of patients with good and poor outcomes by model-based scores. Numbers in parenthesis representing the percentage of patients with poor outcomes; mRS, modified Rankin Scale.

Patients with poor outcomes tended to have a higher score (scores 7–9, 80.0, 68.4 and 57.5% of scores 6, 5, and 4, respectively) (Fig 1). Less than half of the patients with scores 0–3 had poor outcomes (32.3, 17.6, 8.3 and 2.4% of patients with scores 3, 2, 1 and 0, respectively). Therefore, an increasing threshold of the scores in discriminating a binary outcome would increase the ability of the scoring system to accurately detect patients with poor outcomes (or sensitivity) but reduce the ability to accurately detect patients with good outcomes (or specificity).

The sensitivity and specificity of detecting the true outcomes that varied upon each cutoff over the 0–10 score range are summarized in S1 Table. Using the cutoff for a poor outcome at a score of 0 would cover all (100%) patients with poor outcomes, whereas it would miss all patients with good outcomes (or 100% false-positive rate or 0% specificity). Increasing the cutoff to scores of 2, 3 and 4 resulted in a monotonically lower sensitivity to 87.6, 77.7 and 60.3% and an increased specificity to 58.3, 75.0 and 88.1%, respectively.

**Fig 2:**
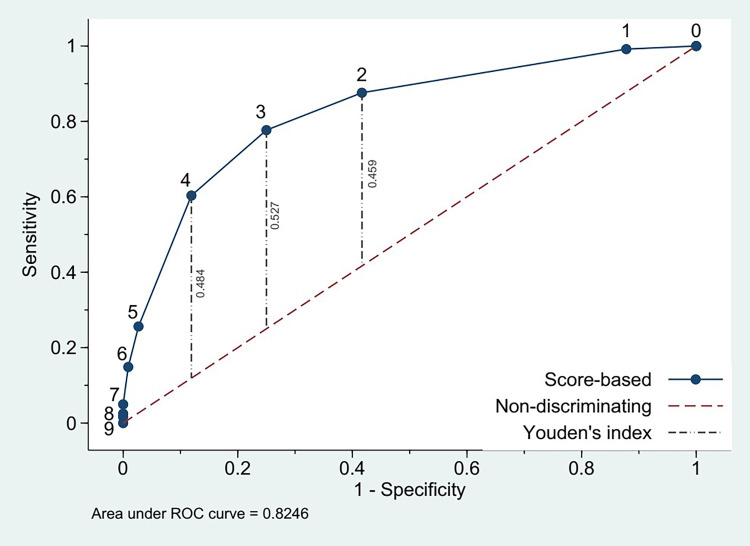
Receiver operating characteristic curve of model-based scores. Youden’s index maximizing summation of sensitivity and specificity and calculated as “sensitivity–(1 –specificity)” was shown for scores 2, 3 and 4, respectively; ROC, receiver operating characteristic.

The area under the ROC curve of the model-based scores was 0.825, indicating an excellent ability to discriminate patients with poor outcomes from those with good outcomes. Youden’s index that maximized the sum of sensitivity and specificity at a given cutoff over the 0–9 score range is illustrated by the vertical distance between the ROC curve and the 45-degree, nondiscriminating line in Fig 2. The index was 0.527, representing the highest performance at the cutoff of 3, followed by 0.484 and 0.459 at the cutoffs of 4 and 2, respectively.

## Discussion

Patients >70 years, having an underlying DM, and presenting with a WFNS scale of IV-V, mFisher scale of 3–4, preoperative rebleeding, and hydrocephalus had a significantly increased risk of combined dependency and death within one year after treatment for aSAH. Based on the risk score ranging from 0 to 10, which was calculated from the summation of the individual scores of 2, 1, 2, 1, 3 and 1 for the presence of the six corresponding characteristics, a score of 3 was an appropriate threshold for discriminating patients with poor outcomes from those with good outcomes.

Other scoring systems have been created to predict poor outcomes in patients with aSAH using preoperative factors [9–11]. The risk factors that were retained in the present scoring system were in line with those reported in previous studies (Table 4). Our study was an incremental step to gain insight into a predictive model suitable for Thailand, where aneurysmal treatment is often delayed and the majority of patients have preexisting hypertension [10, 11, 14] that was poorly controlled [29] and poor adherence to antihypertensive drugs is common [30].

**Table 4:** Published scoring systems for the prediction of poor outcome in patients with aneurysmal SAH.

	Our study n = 457	Mocco et al., 2006 [9] n = 98	Van Donkelaar et al., 2015 [8] n = 1215	Hostettler et al., 2020 [11] n = 621
Country	Thailand	USA	The Netherlands	Switzerland
Clinical and treatment conditions				
rWFNS or Hunt-and-Hess* grading of 4–5	33%	100%*	27%	28%
Fisher or mFisher* grading	3* = 47%	3 = 46%	3 = 10%	3 = 96.1%
	4* = 33%	4 = 37%	4 = 60%	4 = 1.2%
Aneurysmal treatments	Surgery 92%	Surgery 64%	Surgery 41%	Surgery 52%
	Endovascular 8%	Endovascular 36%	Endovascular 44%	Endovascular 47%
Measures of poor outcome	mRS score (1 year), 4–6	mRS score (1 year), 4–6	mRS score (2 months), 4–6	GOS score (1 year), 1–3
Predictors				
Age (years)	>70 = 2 points	>65 = 2 points	50–60 = 1 point	>60 = 1 point
			60–70 = 2 points	
			≥70 = 5 points	
WFNS or rWFNS grading	rWFNS,	--	rWFNS,	WFNS,
	IV-V = 2 points		II = 2 points	III-IV = 2 points
			III = 3 points	V = 3 points
			IV = 6 points	
			V = 9 points	
Hunt-and-Hess grading	--	5 = 2 points	--	--
DM or hyperglycemia	DM = 1 point	Blood glucose ≥ 200 mg/dl = 1 point	--	--
Fisher or mFisher grading	mFisher, 3–4 = 1 point	--	Fisher, 4 = 2 points	--
BNI grading for SAH	--	--	--	Not visible = 1 point
				10–15 mm = 1 point
				>15 mm = 2 points
Preoperative rebleeding	Present = 3 points	--	--	Present = 3 points
Hydrocephalus	Present = 1 point	--	--	Present = 2 points
Size of aneurysm	--	>13 mm = 2 points	10–19.9 mm = 2 points	--
			≥20 mm = 6 points	
Clipping	--	--	--	Present = 2 points
Scoring systems				
Area under the ROC curve	82.5%	--	90%	83.6%
Possible scores (range)	0–10	0–7	0–22	0–13

BNI, Barrow Neurological Institute; DM, diabetes mellitus; GOS, Glasgow Outcome Scale; mFisher, modified Fisher; mg/dl, milligram per decilitre; mm, millimeters; n, number of study patients; ROC, receiver operating characteristic; rWFNS, resuscitated World Federation of Neurosurgical Societies; SAH, subarachnoid hemorrhage; USA, United States of America.

Old age is an established risk factor for poor outcomes [31]. Elderly patients are more likely to have underlying diseases that may complicate postoperative care. As elderly individuals are often frail, this may hamper proper rehabilitation and result in worsening outcomes. A study involving only patients with ruptured anterior communicating artery aneurysms reported a higher need for permanent cerebrospinal fluid diversion in patients >65 years [32]. Patients >85 years overwhelmingly had poor outcomes despite treatments [33]. However, old age itself should not hinder treatments for patients with aSAH, as there is a proportion with reasonable favorable outcomes [34].

For practical reasons, our study used a history of DM rather than measured hyperglycemia as a predictor. Diabetic patients independent of glycemic control had an increased risk of symptomatic cerebral vasospasm [35]. A recent meta-analysis showed that high glucose levels were prevalent at the time of hospital admission and were associated with poor outcomes [36]. The guidelines have suggested keeping glucose levels within normal limits to prevent overexerting brain metabolism [37]. The initial neurological status is correlated with the outcomes. However, recent clinical studies have shown that post resuscitation clinical grades tend to represent outcomes rather than risk factors [38, 39]. Previous studies used the WFNS scale or Hunt-and-Hess measure to grade clinical severity. The Hunt-and-Hess grading, however, contains certain items measuring subjective characteristics, which may lead to unreliable assessments. The present study utilized WFNS scaling when a patient became stable after adequate neurological resuscitation as per the protocol [15]. Poor initial neurological status does not prohibit treatment; thus, both physicians and relatives must weigh the benefit of the procedure against the risk of a vegetative state.

To measure the SAH and associated intracranial hemorrhage, mFisher scaling, which is better than the original Fisher scaling in predicting poor outcomes, was henceforth used in our study. The Barrow Neurological Institute (BNI) grading system that measured the maximal thickness of SAH across cisterns or fissures was recently utilized in the HATCH scoring system [11]. Ninety-six percent of the study patients in the developmental process of HATCH scoring had a Fisher scale of 3; nonetheless, their BNI grades varied. The BNI grading system did not strongly predict cerebral vasospasm, delayed cerebral ischemia or unfavorable outcomes.

Preoperative rebleeding, the strongest predictor in our study, has been reported by other studies as a very strong and independent predictor of mortality [12, 40] and functional dependency [4, 11, 40]. Rebleeding that can lead to severe brain injuries was reported to have a prevalence varying from 8% to 23% [11, 40, 41]. A relatively low percentage (6%) of rebleeding in our study was probably due to survival bias. At local-level hospitals, a majority of cases are not transferred for definite treatment in cases presenting with rebleeding. A score of 3 was assigned to rebleeding in our study, which was equal to the cutoff for poor outcomes. Therefore, patients’ relatives should be fully informed of a high risk of poor outcomes if rebleeding is present prior to definite treatment.

Hydrocephalus is often encountered with aSAH (6–67%) [1, 6, 11, 14, 42, 43]. Acute hydrocephalus results in an increased intracranial pressure, which may result in a higher WFNS grading [44]. In these severe cases, the cerebrospinal fluid must be promptly drained to control the increased intracranial pressure prior to definite treatment [45]. Our study found hydrocephalus was present in 41.1% of patients, and its association with poor outcomes was comparable to that in other studies [4, 11]. A third of patients with aSAH will need permanent cerebrospinal fluid diversion [43].

Certain limitations of our study exist. Our research was a retrospective analysis of electronic health records and a review of medical charts. The clinical measures, however, were unlikely to be prone to misclassification bias, as the variables were objectively measured for common events, which were mandatorily evaluated by the protocols implemented in our neurovascular center. Data collection was conducted in a university-level hospital with facilities superior to their provincial counterparts. Patients treated in the former setting tended to have a better outcome than the latter. Our reported risk factors and scoring algorithm, which is consistent with those reported in the USA and European countries, could be adopted by international communities. To expand their use most widely, this set of outcome predictors should be addressed in the aSAH treatment guidelines.

The strengths of our study include the study sample, which contained a relatively large number of patients receiving treatments under established protocols in a high-level facility. Apart from the excellent discriminative ability of the model (area under the ROC curve, 0.825), the defined predictors were simple and practical for clinical assessment. Therefore, the proposed 0–10 scoring system, in which a score of 3 was the cutoff point for poor outcomes, can be widely applied. A tool to guide decisions on treatments is necessary for countries with limited resources. In Thailand, only approximately 60 supertertiary or tertiary care hospitals are capable of treating aSAH. Our tool can be adopted by a lower-level facility for making decisions about patient transfer for proper treatment at a higher-level facility.

## Conclusions

A scoring system with excellent discrimination of patients with poor outcomes was developed for aSAH. With a simple algorithm incorporating clinical conditions and severity grading, this system is useful in frontline health facilities.

## Supporting information

S1 Table Ability to discriminate between poor and good outcomes by the score cutoff. (DOCX)

S1 Fig Survival of patients by age groups. (TIF)

S2 Fig Survival of patients by the World Federation of Neurosurgical Societies grading scale (WFNS) after resuscitation. (TIF)

S3 Fig Survival of patients by Modified Fisher scores (MFIS). (TIF)

S1 Dataset Aneurysmal subarachnoid hemorrhage data. (ZIP)
