# Supplementary material for: Preoperative predictors of poor outcomes in Thai patients with aneurysmal subarachnoid hemorrhage
Source: PLoS One. 2022 Mar 15;17(3):e0264844. doi: 10.1371/journal.pone.0264844 (PMC8923474; doi:10.1371/journal.pone.0264844)
Supplement: S1 Table — (DOCX) [file pone.0264844.s001.docx]

**S1 Table. Ability to discriminate between poor and good outcomes by the score cutoff**

| Score cutoff | Sensitivity  (True positive rate) | Specificity  (True negative rate) | 1-Specificity  (False-positive rate) | Youden's index^a^ |
| --- | --- | --- | --- | --- |
| 0 | 1.000 | 0.000 | 1.000 | 0.000 |
| 1 | 0.992 | 0.122 | 0.878 | 0.114 |
| 2 | 0.876 | 0.583 | 0.417 | 0.459 |
| 3 | 0.777 | 0.750 | 0.250 | 0.527 |
| 4 | 0.603 | 0.881 | 0.119 | 0.484 |
| 5 | 0.256 | 0.973 | 0.027 | 0.229 |
| 6 | 0.149 | 0.991 | 0.009 | 0.140 |
| 7 | 0.050 | 1.000 | 0.000 | 0.050 |
| 8 | 0.025 | 1.000 | 0.000 | 0.025 |
| 9 | 0.017 | 1.000 | 0.000 | 0.017 |
| 10 | 0.000 | 1.000 | 0.000 | 0.000 |
| ^a^ sensitivity – (1 – specificity) or sensitivity + specificity – 1 | | | | |
