# Supplementary figures and images for: Preoperative predictors of poor outcomes in Thai patients with aneurysmal subarachnoid hemorrhage
Source: PLoS One. 2022 Mar 15;17(3):e0264844. doi: 10.1371/journal.pone.0264844 (PMC8923474; doi:10.1371/journal.pone.0264844)

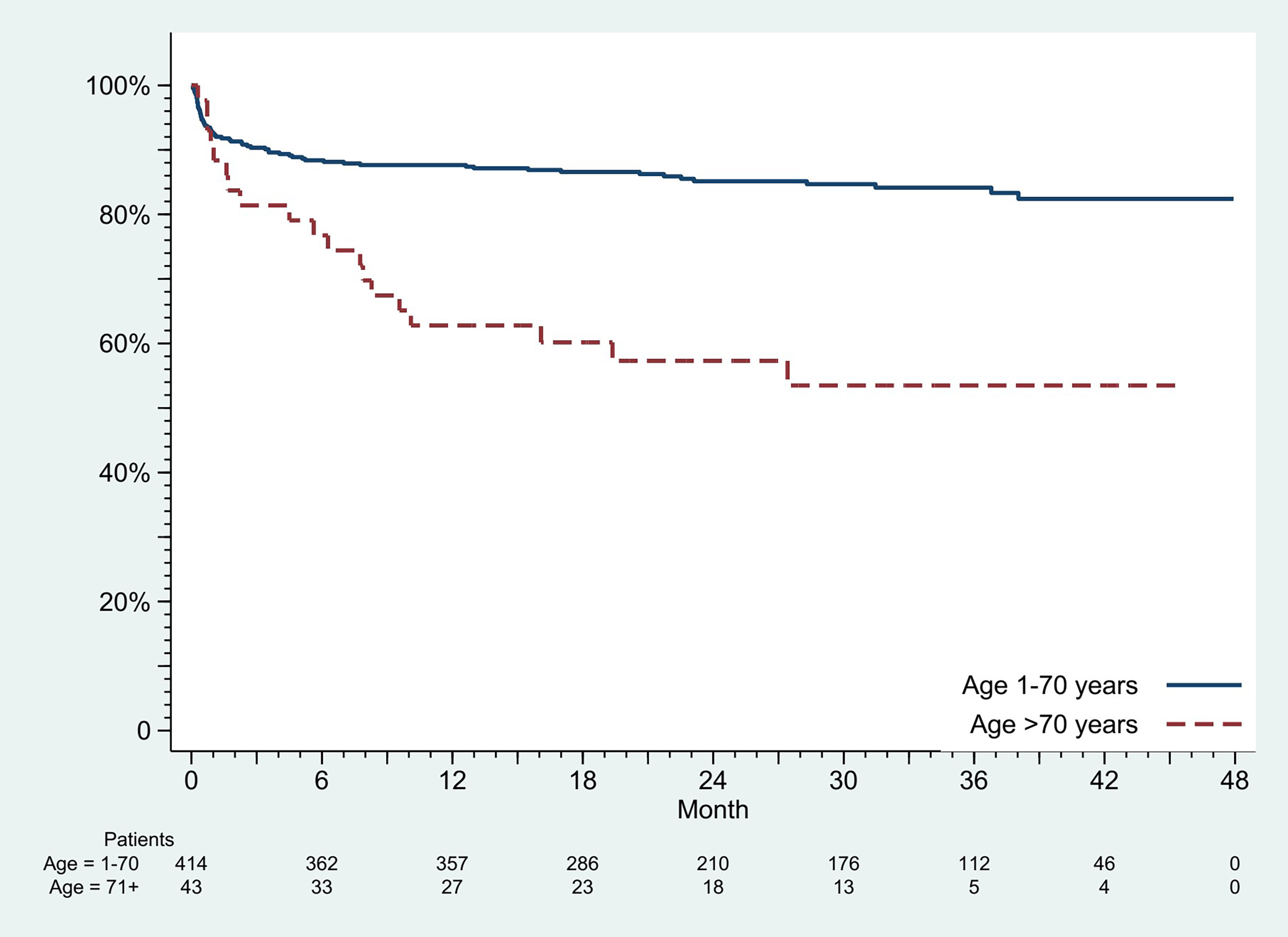

Supplement: S1 Fig — (TIF) [file pone.0264844.s002.tif]

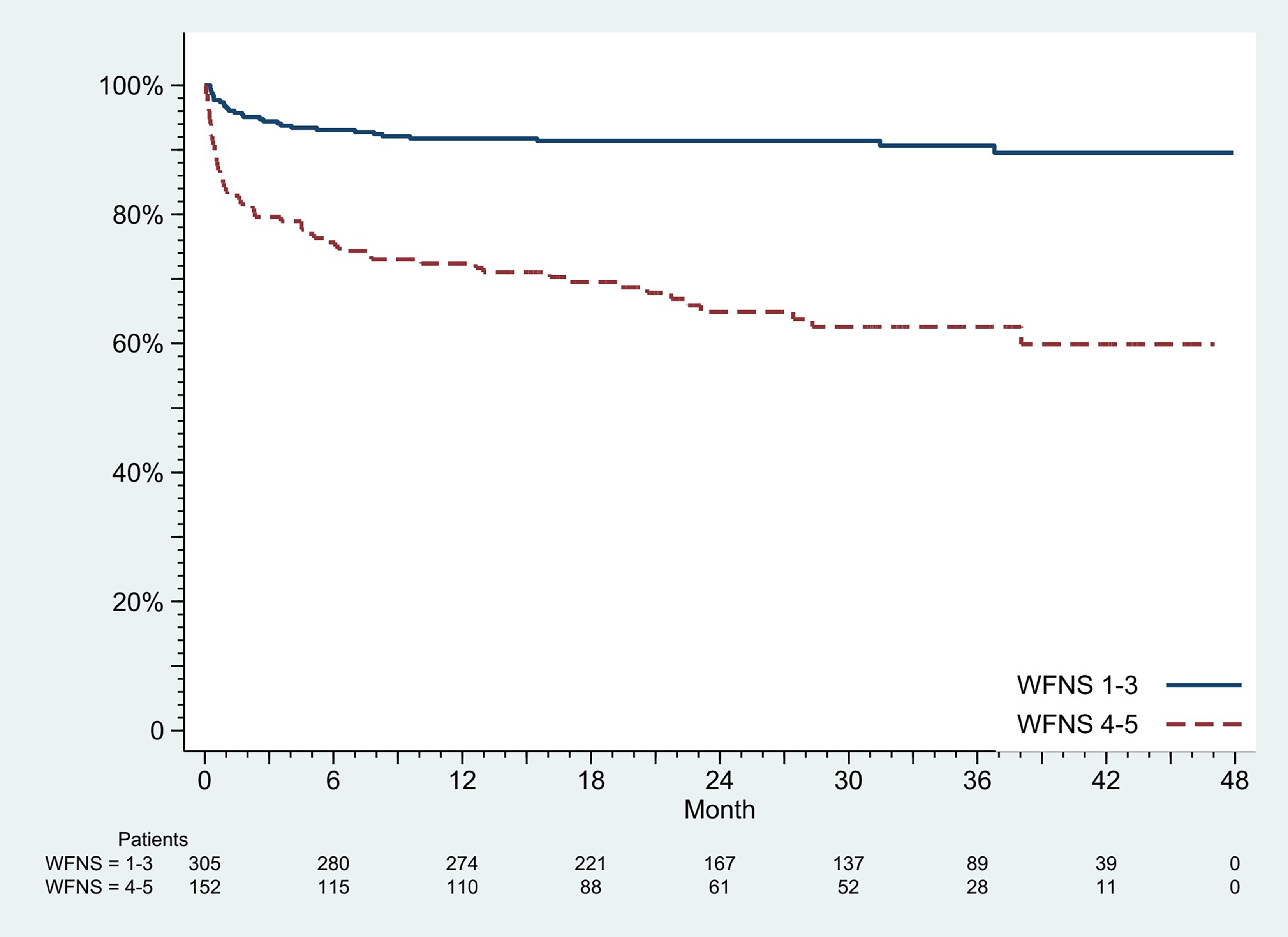

Supplement: S2 Fig — (TIF) [file pone.0264844.s003.tif]

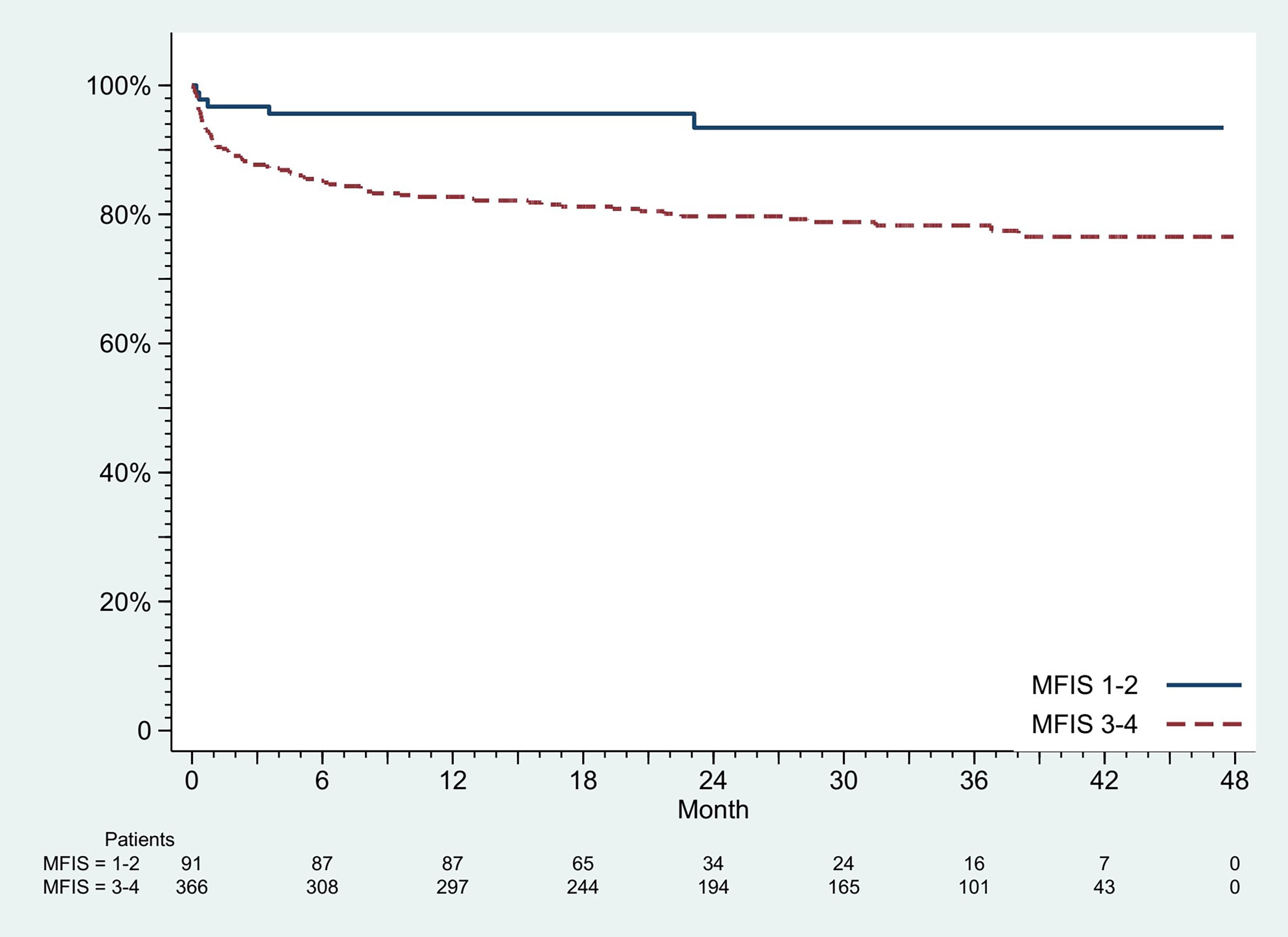

Supplement: S3 Fig — (TIF) [file pone.0264844.s004.tif]
